# Nonexercise estimated cardiorespiratory fitness in relation to incidence of urinary tract, bladder and kidney cancer in the HUNT study

**DOI:** 10.1038/s41598-025-29410-7

**Published:** 2025-11-24

**Authors:** Youssef Khalil, Yi-Qian Sun, Xiao-Mei Mai

**Affiliations:** 1 HUNT Center for Molecular and Clinical Epidemiology, Department of Public Health and Nursing, Norwegian University of Science and Technology, Trondheim, Norway; 2 Department of Neuromedicine and Movement Science, Norwegian University of Science and Technology, Trondheim, Norway; 3 Department of Clinical and Molecular Medicine, Faculty of Medicine and Health Science, Norwegian University of Science and Technology, Trondheim, Norway; 4 Clinic of Laboratory Medicine, Department of Pathology, St. Olavs Hospital, Trondheim University Hospital, Trondheim, Norway; 5 TkMidt-Center for Oral Health Services and Research, Mid-Norway, Trondheim, Norway; 6 Department of Public Health and Nursing, Norwegian University of Science and Technology, Trondheim, Norway

**Keywords:** Bladder cancer, Cardiorespiratory fitness, Estimated cardiorespiratory fitness, Kidney cancer, Physical activity, Urinary tract cancer, Urological cancer, Renal cancer, Renal cancer, Urological cancer, Epidemiology, Risk factors, Bladder, Epidemiology

## Abstract

This study examined the prospective relationships between estimated cardiorespiratory fitness (eCRF) and the incidence of urinary tract cancers, as well as the site-specific incidence of bladder cancer and kidney cancer within a large Norwegian cohort. We included 46,968 cancer-free adults from the second Trøndelag Health Study (HUNT2). eCRF was determined using sex-specific prediction models based on age, waist circumference, resting heart rate, and self-reported physical activity, and further classified into three categories: 20% low, 40% medium, and 40% high. Cause-specific Cox proportional hazard models were applied to estimate hazard ratios (HR) and 95% confidence intervals (CI). Over a median follow-up of 22.2 years, medium and high eCRF were associated with a 13% and 36%, respectively, lower hazard of urinary tract cancers in the total cohort (HR 0.87, 95% CI 0.71–1.05 and HR 0.64, 95% CI 0.51–0.79). Among men, these associations were more pronounced, with a hazard reduction of 17% and 41% in the medium and high eCRF groups, respectively (HR 0.83, 95% CI 0.66–1.04 and HR 0.59, 95% CI 0.46–0.76). High eCRF only was associated with a 34% lower hazard of bladder cancer in men (HR 0.66, 95% CI 0.48–0.90), but not in women. There was a significant inverse dose-response association between eCRF and kidney cancer, particularly among men (*P*-value for trend < 0.001). In conclusion, eCRF may be a useful marker for evaluating the associations between CRF and urinary tract cancers.

**Supplementary Information:**

The online version contains supplementary material available at 10.1038/s41598-025-29410-7.

## Introduction

Urinary tract cancers, including bladder cancer and kidney cancer, represent a significant health burden globally. There were 1.05 million new cases of these forms of cancers in 2022, with new cases projected to increase by 72% in 2050^1,2^. Multiple modifiable risk factors may influence the increasing incidence of these cancers, including obesity, physical inactivity, smoking, alcohol consumption, hypertension, diabetes mellitus, and occupational hazards^3,4^. Since these cancers disproportionately affect men^2,3^, it is important to study potential differences in risk factors between men and women to improve sex-specific prevention strategies.

Previous literature suggested that positive lifestyle changes could potentially prevent 33–50% of cancers^5^. For instance, systematic review and meta-analysis studies showed that higher physical activity (PA) levels may reduce risks of bladder cancer and kidney cancer by 15% and 12%, respectively^6,7^. However, reliance on self-reported PA introduces potential biases such as recall bias, underscoring the need for more accurate fitness measures^8^.

Cardiorespiratory fitness (CRF) is an objective marker of overall health^9^. CRF is optimally assessed by measuring the individual’s maximal oxygen uptake (VO_2max_) through exercise. Higher exercise-measured CRF levels are consistently linked to reduced risks of all-cause mortality^10–12^, cancer-specific mortality^12,13^, as well as cancer incidence^13–17^. For example, a Swedish cohort of over 1 million young men found a 20% reduction in the incidence of kidney cancer and a potential reduction in the incidence of bladder cancer among those with higher CRF levels^17^. Similarly, a Norwegian study showed an inverse association between CRF levels and risks of bladder cancer, pancreatic cancer, and lung cancer, but not with kidney cancer in men^16^. However, these studies focused only on men and lacked adjustment for socioeconomic factors as a potential confounder^16,17^. Additionally, exercise-measured CRF is a resource-intensive measurement, requiring specialized equipment, trained personnel, and strict protocols, which limits their feasibility for large-scale or older populations^18,19^.

To address these challenges, estimated CRF (eCRF) has emerged as a non-exercise-based alternative for predicting peak oxygen uptake (VO_2peak_)^18,20^. Estimated CRF offers a cost-effective and practical method for assessing fitness in large-population studies and has recently been approved by the American Heart Association^21,22^. Limited research has explored the relationship between eCRF and site-specific cancer incidence, suggesting inverse associations with prostate cancer and colorectal cancer in men and breast cancer in women^18,22^. To our knowledge, no prior studies have examined the association between eCRF and the incidence of urinary tract cancers.

Therefore, our study aimed to examine the prospective relationships between CRF, estimated from non-exercise prediction models, and: (1) the incidence of urinary tract cancers overall, as well as (2) the site-specific incidence of bladder cancer and kidney cancer in a large population-based survey.

## Results

### Baseline characteristics

The mean age was 47.3 years (standard deviation [SD] = 16.0) for men and 46.4 years (SD = 16.4) for women. The baseline characteristics by eCRF categories in men and women, respectively, are presented in Table 1 and Supplementary Table 1. Overall, participants in higher eCRF categories were younger, more physically active, and more classified into the higher occupational classes; they had a lower prevalence of obesity and smoking and higher education levels. These patterns were consistent across men and women.

**Table 1:** Baseline characteristics of 46,968 participants in the HUNT Study, stratified by eCRF categories.

	eCRF categories in men (*n* = 23,375)			eCRF categories in women (*n* = 23,593)		
	20% low	40% medium	40% high	20% low	40% medium	40% high
N	4676	9351	9348	4721	9437	9435
Age (years)	48.4 (16.3)	47.6 (15.9)	46.4 (15.9)	47.6 (16.8)	46.8 (16.5)	45.3 (16.2)
WC (cm)	102.5 (8.7)	92.5 (6.1)	84.9 (6.0)	94.3 (10.6)	80.9 (8.0)	72.9 (6.4)
Body mass index (kg/m²)	30.8 (5.0)	25.9 (3.5)	23.4 (2.7)	29.9 (3.7)	26.6 (2.7)	24.4 (2.4)
RHR (bmp)	79.4 (13.3)	71.2 (10.9)	63.6 (9.8)	81.6 (13.6)	75.5 (11.4)	69.0 (10.0)
eCRF (mL/kg/min)	37.9 (7.3)	44.2 (6.6)	49.7 (6.6)	30.0 (6.6)	35.1 (6.2)	39.4 (5.9)
Meet the ACSM’s recommendations for physical activity						
No	3509 (75.0%)	4554 (48.7%)	1600 (17.1%)	3635 (77.0%)	5373 (56.9%)	1841 (19.5%)
Yes	1167 (25.0%)	4797 (51.3%)	7748 (82.9%)	1086 (23.0%)	4064 (43.1%)	7594 (80.5%)

ACSM, American College of Sports Medicine; eCRF, estimated cardiorespiratory fitness.

Data: as means (standard deviation) for continuous variables and participant counts (percentages) for categorical variables.

eCRF is stratified by sex and age-specific categories: 20% in the low category, 40% in the medium category, and 40% in the high category.

### Association between eCRF and the incidence of urinary tract cancers

During a median follow-up period of 22.2 years, 652 urinary tract cancer cases were observed, 468 in men and 184 in women. Among the entire study population, the high eCRF group had an HR of 0.64 (95% CI 0.51–0.79), and the medium eCRF group had an HR of 0.87 (95% CI 0.71–1.05) for the urinary tract cancers, with a *P*-value for trend < 0.001 (Table 2). When stratified by sex, men in the medium and high eCRF categories had a respectively 17% and 41% reduced hazard of urinary tract cancers (HR 0.83, 95% CI 0.66–1.04, and HR 0.59, 95% CI 0.46–0.76, *P*-value for trend < 0.001). Among women, the association appeared weaker (*P*-value for trend = 0.12), but there was no evidence of effect modification by sex (*P*-value for likelihood ratio test (LRT) = 0.48). Cumulative cause-specific hazard curves by eCRF category are presented in Supplementary Fig. 1.

**Table 2:** The association between eCRF categories and the incidence of urinary tract cancers in HUNT study.

eCRF categories	Cases	Incidence rate (per 1000 person-years)	Crude model			Model 1			Model 2		
			HR (95% CI)		*P*-value for trend	HR (95% CI)		*P*-value for trend	HR (95% CI)		*P*-value for trend
Total (*n* = 46,968)											
20% low (*n* = 9397)	161	0.88	1.00	[Reference]	< 0.001	1.00	[Reference]	< 0.001	1.00	[Reference]	< 0.001
40% medium (*n* = 18,788)	287	0.76	0.85	(0.70–1.03)		0.87	(0.71–1.05)		0.83	(0.70–1.03)	
40% high (*n* = 18,783)	204	0.53	0.60	(0.48–0.73)		0.64	(0.51–0.79)		0.61	(0.46–0.80)	
Men (*n* = 23,375)											
20% low (*n* = 4676)	120	1.35	1.00	[Reference]	< 0.001	1.00	[Reference]	< 0.001	1.00	[Reference]	< 0.001
40% medium (*n* = 9351)	206	1.12	0.80	(0.64–1.00)		0.83	(0.66–1.04)		0.78	(0.61–1.01)	
40% high (*n* = 9348)	142	0.75	0.54	(0.42–0.69)		0.59	(0.46–0.76)		0.55	(0.40–0.76)	
Women (*n* = 23,593)											
20% low (*n* = 4721)	41	0.43	1.00	[Reference]	0.11	1.00	[Reference]	0.12	1.00	[Reference]	0.41
40% medium (*n* = 9437)	81	0.42	0.96	(0.66–1.40)		0.95	(0.65–1.39)		0.99	(0.65–1.52)	
40% high (*n* = 9435)	62	0.31	0.74	(0.50–1.10)		0.74	(0.49–1.11)		0.82	(0.48–1.39)	

CI, confidence interval; eCRF, estimated cardiorespiratory fitness; HR, hazard ratio. eCRF is stratified by sex and 10-year age-specific categories: 20% in the low category, 40% in the medium category, and 40% in the high category.

Crude model: Age is used as the time scale.

Model 1: Age is used as the time scale and adjusted for sex (only in total cohort), sitting time, smoking, alcohol consumption, education, occupational class, family history of cancer, hypertension, and diabetes.

Model 2: Adjusted for body mass index and physical activity in addition to variables in Model 1.

### Association between eCRF and the incidence of bladder cancer and kidney cancer

A total of 381 bladder cancer cases (299 in men and 82 in women) and 255 kidney cancer cases (160 in men and 95 in women) were identified. Because of the observed effect modification by sex for bladder cancer (*P*-value for LRT = 0.03), we presented the results separately for men and women and not in the total population (Table 3). Among men, only the high eCRF was associated with a reduced incidence of bladder cancer (HR 0.66, 95% CI 0.48–0.90), while no association was observed in women (HR 0.93, 95% CI 0.52–1.66).

For kidney cancer, those with medium and high levels of eCRF in the total cohort demonstrated HRs of 0.81 (95% CI 0.60–1.10) and 0.51 (95% CI 0.36–0.72) (*P*-value for trend < 0.001) (Table 4), respectively. In men, both medium and high eCRF categories were associated with reduced incidence of kidney cancer, showing a dose-response association (*P*-value for trend < 0.001). Among women, although only the high eCRF category was associated with a significant reduction in the incidence of kidney cancer, the LRT did not indicate a statistically significant effect modification by sex (*P*-value = 0.08).

**Table 3:** The association between eCRF categories and the incidence of bladder cancer in HUNT study.

eCRF categories	Cases	Incidence rate (per 1000 person-years)	Crude model		*P*-value for trend	Model 1			Model 2		*P*-value for trend
			HR (95% CI)			HR (95% CI)		*P*-value for trend	HR (95% CI)		
Men (*n* = 23,375)											
20% low (*n* = 4676)	71	0.80	1.00	[Reference]	< 0.001	1.00	[Reference]	0.006	1.00	[Reference]	0.09
40% medium (*n* = 9351)	135	0.73	0.88	(0.66–1.17)		0.92	(0.69–1.23)		0.92	(0.67–1.27)	
40% high (*n* = 9348)	93	0.49	0.59	(0.43–0.80)		0.66	(0.48–0.90)		0.72	(0.48–1.07)	
Women (*n* = 23,593)											
20% low (*n* = 4721)	18	0.19	1.00	[Reference]	0.63	1.00	[Reference]	0.87	1.00	[Reference]	0.64
40% medium (*n* = 9437)	26	0.13	0.70	(0.39–1.28)		0.64	(0.35–1.18)		0.51	(0.26–1.00)	
40% high (*n* = 9435)	38	0.19	1.03	(0.59–1.81)		0.93	(0.52–1.66)		0.70	(0.33–1.50)	

CI, confidence interval; eCRF, estimated cardiorespiratory fitness; HR, hazard ratio. eCRF is stratified by sex and 10-year age-specific categories: 20% in the low category, 40% in the medium category, and 40% in the high category.

Crude model: Age is used as the time scale.

Model 1: Age is used as the time scale and adjusted for sitting time, smoking, alcohol consumption, education, occupational class, family history of cancer, hypertension, and diabetes.

Model 2: Adjusted for body mass index and physical activity in addition to variables in Model 1.

**Table 4:** The association between eCRF categories and the incidence of kidney cancer in HUNT study.

eCRF categories	Cases	Incidence rate (per 1000 person-years)	Crude model		*P*-value for trend	Model 1		*P*-value for trend	Model 2		*P*-value for trend
			HR (95% CI)			HR (95% CI)			HR (95% CI)		
Total (*n* = 46,968)											
20% low (*n* = 9397)	71	0.39	1.00	[Reference]	< 0.001	1.00	[Reference]	< 0.001	1.00	[Reference]	0.001
40% medium (*n* = 18,788)	115	0.30	0.78	(0.58–1.05)		0.81	(0.60–1.10)		0.80	(0.57–1.13)	
40% high (*n* = 18,783)	69	0.18	0.47	(0.33–0.65)		0.51	(0.36–0.72)		0.46	(0.30–0.72)	
Men (*n* = 23,375)											
20% low (*n* = 4676)	49	0.55	1.00	[Reference]	< 0.001	1.00	[Reference]	0.001	1.00	[Reference]	< 0.001
40% medium (*n* = 9351)	64	0.35	0.63	(0.43–0.91)		0.63	(0.43–0.92)		0.53	(0.35–0.82)	
40% high (*n* = 9348)	47	0.25	0.45	(0.30–0.68)		0.49	(0.32–0.74)		0.32	(0.19–0.56)	
Women (*n* = 23,593)											
20% low (*n* = 4721)	22	0.23	1.00	[Reference]	0.008	1.00	[Reference]	0.02	1.00	[Reference]	0.50
40% medium (*n* = 9437)	51	0.26	1.13	(0.68–1.86)		1.17	(0.71–1.95)		1.48	(0.84–2.62)	
40% high (*n* = 9435)	22	0.11	0.49	(0.27–0.88)		0.52	(0.28–0.96)		0.79	(0.36–1.71)	

eCRF is stratified by sex and 10-year age-specific categories: 20% in the low category, 40% in the medium category, and 40% in the high category.

Crude model: Age is used as the time scale.

Model 1: Age is used as the time scale and adjusted for sex (only in total cohort), sitting time, smoking, alcohol consumption, education, occupational class, family history of cancer, hypertension, and diabetes.

Model 2: Adjusted for body mass index and physical activity in addition to variables in Model 1.

## Sensitivity Analysis

After further adjustment for BMI and PA in Model 2, the associations between eCRF categories and the incidence of urinary tract cancers, bladder cancer, and kidney cancer did not change substantially in the total cohort and by sex (Tables 2, 3, and 4). After excluding the first three years of the follow-up period, the results for the associations between eCRF categories and incidence of urinary tract cancers (n=605 cases), bladder cancer (n=350 cases), and kidney cancer (n=250 cases) remained largely consistent with the main results in the total cohort, men and women (Supplementary Tables 2, 3, and 4), except that the HR between high eCRF and kidney cancer became somewhat imprecise among women (from HR 0.52, 95% CI: 0.28–0.96 to HR 0.55,95% CI: 0.29–1.04, Supplementary Table 4). Also, the associations between the eCRF in tertiles and the incidence of urinary tract cancers, bladder cancer, and kidney cancer were similar to the main results, where eCRF was classified as 20% low, 40% medium, and 40% high (Supplementary Tables 5, 6, and 7). For the results of the multiple imputations (Supplementary Tables 8, 9, and 10), the HRs of the associations between eCRF and all cancer types did not change from the main analyses, where confounders with missing values were assigned to a separate “unknown” category. Finally, in the sensitivity analyses using time-varying covariates(Supplementary Tables 11, 12, and 13), the HRs for the incidence of urinary tract cancers and kidney cancer were similar to those in the main analyses. However, for bladder cancer in men, the association with high eCRF was slightly weaker, with the HR changing from the original 0.66 to 0.72(Supplementary Table 12).

## Discussion

To our knowledge, this is one of the first large-scale prospective studies to examine the association between eCRF and the incidence of urinary tract cancers, and among the first to include women. We found that eCRF was inversely associated with the incidence of urinary tract cancers and the site-specific incidence of kidney cancer in a dose-response manner in the total cohort, and particularly among men. Our analysis also revealed that the high eCRF level only was associated with a lower incidence of bladder cancer in men but not in women.

Previous studies investigated the relationship between eCRF and the overall incidence of cancer^18,22^. Wang et al.^18^ revealed a clear inverse dose-response relationship between eCRF and overall incidence of cancer in the total cohort and especially among men. As is the case with the current study, they did not observe an effect modification by sex. Similarly, the NIH-AARP Diet and Health Study found an inverse graded relationship for both sexes in a cohort of 402,548 adults with a mean age of 62 years^22^. The unified association between men and women observed in the NIH-AARP Study could be due to their older cohort, which likely resulted in more cancer events and increased statistical power.

In the current study, we observed that the high eCRF level only was associated with a 34% reduced incidence of bladder cancer in men, while no association was observed in women. Prior studies investigated the relationship between exercise-measured CRF and the incidence of bladder cancer only among men^16,17^. Onerup et al.^17^ reported a 10% reduction in the incidence of bladder cancer among men with high CRF in a large cohort, while Robsahm et al.^16^ found a 60% lower incidence among men in the highest CRF tertile. Differences in the magnitude of the association estimates may be due to demographic variations; for instance, Onerup et al.^17^ included younger men aged 16–25 years, whereas we included a relatively older population with a mean age of 46.8 years. The study by Robsahm et al.^16^ was relatively small and may be subjected to selection bias. Furthermore, the different CRF classifications used across these studies could lead to varied association estimates. However, using two distinct classifications in our analysis yielded consistent findings. Unlike previous studies^16,17^, which did not account for confounders like smoking, alcohol consumption, or socioeconomic status, our rigorous adjustment for these factors might provide more accurate estimates. Given that previous research often overlooked women^16,17^, larger studies including women are needed to explore the sex-specific impact of eCRF on the incidence of bladder cancer and to validate our findings.

The link between exercise-measured or estimated CRF and the incidence of kidney cancer has not been well studied, especially among women. Our study addressed this gap by reporting a 48% reduction in the incidence of kidney cancer among women with high eCRF. Additionally, we observed an inverse dose-response relationship where medium and high eCRF were associated with a 19% and 49%, respectively, reduction in the incidence of kidney cancer across the total cohort, and 37% and 51% reduction among men. This was generally in line with the study by Onerup et al.^17^, who reported reduced hazards of 8% for medium and 20% for high exercise-measured CRF. In contrast to our findings and those of Onerup et al.^17^, Robsahm et al.^16^ did not report any significant association between CRF tertiles and the incidence of kidney cancer. This might be explained by the same reasons mentioned earlier for bladder cancer.

The biological mechanisms underlying the role of CRF in reducing the incidence of urinary tract cancers remain unclear. As CRF represents the overall health^9^, optimal CRF may protect against cancer by reducing systemic inflammation and oxidative stress, boosting immune responses, preventing DNA mutations, and enhancing DNA repair processes^6,23^. Higher CRF may directly improve key metabolic pathways involved in cell proliferation and apoptosis^23^. Furthermore, higher CRF is often associated with decreased adiposity, which may indirectly reduce the incidence of cancer through its beneficial effects on insulin sensitivity^23^.

Some biological mechanisms may explain the observed sex differences in the incidence of bladder cancer^24^. For example, men may have less efficient metabolism of carcinogens, such as tobacco-derived aromatic amines that damage DNA. As a result, the male urothelium may be more exposed to these carcinogens, contributing to the higher incidence of bladder cancer in men compared to women^24^. However, it is possible that the difference in carcinogen metabolism among men with high versus low CRF could be more pronounced than the corresponding difference among women. This might explain why we observed an association between high CRF and reduced incidence of bladder cancer in men but not in women. Further research is warranted to explore the biological mechanisms through which CRF may protect against urinary tract cancers and to better understand sex-based differences in the association between CRF and the incidence of bladder cancer.

Since previous studies were conducted mainly in men, the novelty of this study lies in examining these associations additionally in women with a long follow-up period. The study population is drawn from a region with predominantly native inhabitants. Our algorithms for calculating eCRF are derived from the same source of population, allowing for better estimation^20^. We have also adjusted for a comprehensive panel of potential confounders, such as lifestyle and socioeconomic factors, enhancing our study’s internal validity. Cancer diagnoses are obtained from the Cancer Registry of Norway, ensuring the accuracy of cancer outcomes.

Nonetheless, the present study has some limitations. First, excluding participants with missing data on waist circumference (WC), resting heart rate (RHR), or PA may have introduced selection bias and led to a possible underestimation of the true association, especially in women, since those excluded were generally older, being women, or less educated^18^. Hence, the results should be interpreted with caution. Second, using predictive models for estimating CRF might introduce a non-differential misclassification bias in the current study, that is, overestimating CRF for the least active and underestimating it for the most active^25^. Despite this, Nes et al.^25^ showed that these models could identify those at increased risk for chronic diseases, enabling them to benefit from preventive interventions. Third, eCRF was calculated only once at baseline, limiting the ability to account for changes in eCRF throughout the study period. Fourth, residual confounding cannot be ruled out due to missing data on diet and occupational exposure. However, the HUNT population is derived from a region with limited high-risk occupations. Regarding adjustment, we did not include BMI and PA in the main Models. As Garnvik et al.^20^ recommended, future studies using similar eCRF equations should consider excluding these variables from adjustment to avoid over-adjustment. Fifth, our study was limited by a small sample size for some of the associations, especially in women. Lastly, extrapolating our results to populations with other ethnicities should be done with caution.

In conclusion, we observed that higher eCRF levels were inversely associated with the incidence of urinary tract cancers and the site-specific incidence of kidney cancer in the total cohort and especially among men. High eCRF only was inversely associated with the incidence of bladder cancer in men but not in women. Estimated CRF may be a useful marker for evaluating the associations between CRF and urinary tract cancers. Given that CRF can be estimated in routine clinical examinations, it may support population-level risk stratification and cancer prevention strategies.

## Methods

### Study design and population

This prospective study utilized data from the Trøndelag Health Study (HUNT), Norway’s largest population-based survey^26^. Details on the HUNT study have been described elsewhere^26^. Briefly, HUNT encompasses four phases (HUNT1: 1984–1986, HUNT2: 1995–1997, HUNT3: 2006–2008, and HUNT4: 2017–2019), collecting health data through self-reported questionnaires, clinical examinations, and laboratory tests^26^. In this study, we adhered to the guidelines outlined in the Strengthening the Reporting of Observational Studies in Epidemiology (STROBE)^27^.

**Fig. 1:**
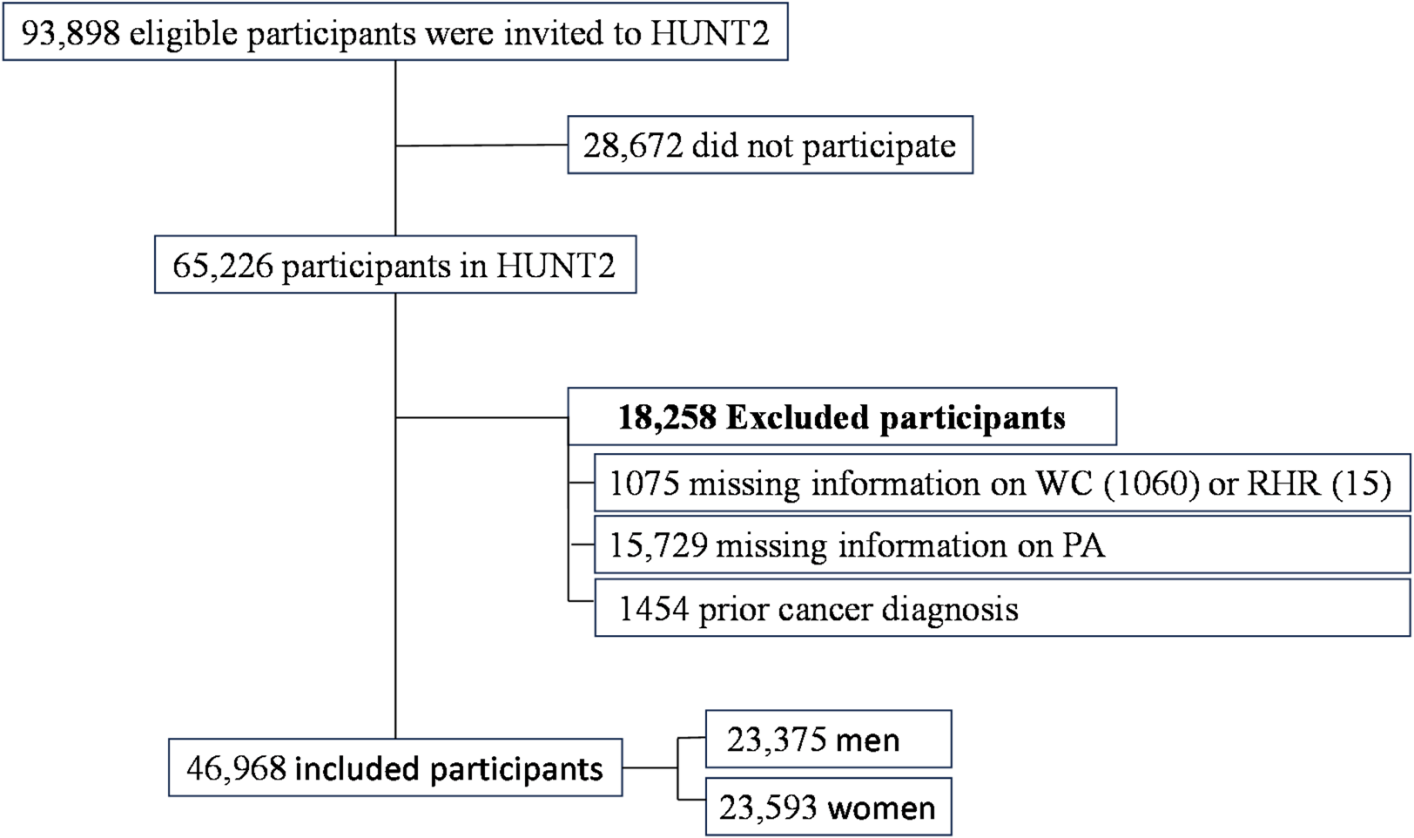
The flowchart illustrates the exclusion criteria for participants in this study. Participants were excluded mainly due to a lack of data on waist circumference (WC), resting heart rate (RHR), or physical activity (PA), or prior cancer diagnosis at baseline.

Data from HUNT2 (1995–1997) were used, where 93,898 eligible participants aged ≥ 20 were invited. Out of these, 65,226 (69.5%) individuals participated. First, we excluded 1075 participants without information on WC (*n* = 1060) or RHR (*n* = 15). Second, we excluded those who had missing information on PA (*n* = 15,729). Lastly, participants with prior cancer diagnoses (*n* = 1454) were excluded. Consequently, the final analysis comprised 46,968 cancer-free participants, including 23,375 (49.8%) men and 23,593 (50.2%) women (Fig. 1).

### Estimated CRF

Estimated CRF was calculated from non-exercise prediction models. The models aimed at predicting eCRF in VO_2peak_ (ml/kg/min) and were derived from a sample of healthy middle-aged adults from the HUNT3 population^25^ and further adapted for use in HUNT2^21^. The variables included in the prediction models were age, WC, RHR, and self-reported PA. The models and the self-reported PA questionnaires were previously validated^21,25,28^, suggesting high comparability with other non-exercise prediction models^25,29,30^.

The prediction models used for estimating CRF are as follows^25^:

For men (Goodness of fit statistic = 0.58, Standard error of estimate = 5.88):

$$105.91 -(0.334 \times \mathrm{Age}) - (0.402 \times \mathrm{WC}) - (0.144 \times \mathrm{RHR}) + (3.102 \times \mathrm{PA}_{\mathrm{ACSM}}\bigr)$$

For women (Goodness of fit statistic = 0.52, Standard error of estimate = 5.37):

$$78.00 - (0.297 \times \mathrm{Age}) - (0.270 \times \mathrm{WC}) - (0.110 \times \mathrm{RHR}) + (2.674 \times \mathrm{PA}_{\mathrm{ACSM}}\bigr)$$

To measure WC, a metal tape was used at the participants’ umbilicus level while standing with the arms flattened and registered to the nearest centimeter^18,31^. RHR was calculated as the mean of three measurements recorded after sitting for two minutes using Dinamap 845XT (Critikon). Data on PA was obtained from the self-reported questionnaires. Participants answered questions about their intensity levels and duration of leisure-time PA during the past year. Intensity was divided into: light, defined as “not sweating/being out of breath” and vigorous, defined as “sweating/out of breath”. The duration of each intensity level was reported as an average of hours per week (0, < 1 h, 1–2 h, or ≥ 3 h). We initially classified participants’ levels of PA into four categories: inactive (no activity, or ≤ 2 h light activity only), low (≥ 3 h light activity only, or ≤ 2 h light activity and < 1 h vigorous activity), moderate (≥ 3 h light activity and < 1 h vigorous activity, or 1–2 h vigorous activity regardless of light activity), and high (≥ 3 h vigorous activity regardless of light activity)^32^. This classification was widely used in studies utilizing HUNT2 and showed a dose-response association of PA levels with overall cancer incidence and mortality^18,32^.

To calculate eCRF, we further classified PA into two categories (PA_ACSM_): a value of “1” was given to those who had moderate or high PA levels. They were regarded as meeting the American College of Sports Medicine (ACSM) recommendations, i.e., at least 75 min (min) per week of vigorous intensity exercise or at least 150 min per week of moderate intensity exercise; and a value of “0” was given to those who were inactive or had low PA level. They were regarded as not meeting the ACSM recommendations^21^.

Estimated CRF was further classified into age-specific (based on a 10-year interval) quintiles within each sex group to account for the possible curvilinear association between age and CRF and reduce age-related misclassification^33^. This approach, recommended by Kokkinos et al. ^33^, helps minimize variability in both mean age and sample size across fitness categories and, therefore, improve the validity of associations with health outcomes. Then, we merged the quintiles into three broader groups: the first quintile as the 20% low eCRF, the second and third quintiles as the 40% medium eCRF, and the fourth and fifth quintiles as the 40% high eCRF group. This reclassification was derived from the Aerobics Center Longitudinal Study (ACLS) and applied here to optimize statistical power^34^. We have also classified eCRF into age and sex-specific tertiles to be consistent with other previous literature and to assess the robustness of our results from the ACLS classification^16,18^.

### Follow-up and the assessment of the outcome

The HUNT2 data were linked with the Cancer Registry of Norway using the personal identification number. The cancer outcomes were identified using the International Statistical Classification of Disease and Related Health Problems (ICD-10)^35^. Urinary tract cancers, bladder cancer, and kidney cancer were coded as (C64–C68), (C67), and (C64), respectively. The follow-up period for participants started from baseline until one of the following occurred: first diagnosis of any of these cancers, death, emigration from the county, or the year-end of 2018.

### Statistical analysis

Baseline characteristics across the three age-specific eCRF categories were presented separately for men and women using descriptive statistics: means (standard deviations) for continuous variables and counts (percentages) for categorical variables. We used cause-specific Cox proportional hazards (PH) models to assess the relationships between eCRF and the incidence of urinary tract cancers, along with the site-specific incidence of bladder cancer and kidney cancer^36,37^. By employing cause-specific hazard models, we aimed to evaluate a potentially etiological association of eCRF with the instantaneous rate of cancer occurrence among individuals who remained event-free over time. Crude (age as the timescale) and adjusted hazard ratios (HRs) with 95% confidence intervals (CIs) were calculated in three models separately (Crude model, Model 1, and Model 2), with low eCRF being the reference group. In addition to age in the Crude model, Model 1 adjusted for sex (in total cohort only), sitting time, smoking, alcohol consumption, education, occupational class, family history of cancer, hypertension, and diabetes mellitus. Model 2 was further adjusted for body mass index (BMI) and PA as a part of the sensitivity analysis, as WC and PA were already included in the eCRF Equation^20^. Details on variable classifications are provided in the Supplementary Methods. Hazard ratios from Model 1 were used as the primary estimates. Analyses were conducted for the total cohort and stratified by sex. To assess trends across eCRF categories, we included eCRF as an ordinal variable in the Cox regression models and reported the corresponding *P*-values for trend in all the models. We also generated cumulative hazard curves stratified by eCRF categories, based on the cause-specific Cox models, to visually illustrate differences in the rate of event occurrence over time^36^. Missing data on confounders were handled using the missing-indicator method by assigning a separate “unknown” category in the main analyses.

PH assumptions were tested using Schoenfeld residuals, and there was no evidence of potential violations. To test effect modification by sex in the association between eCRF and the incidence of urinary tract cancers, bladder cancer, or kidney cancer, we compared the Cox PH models with and without including an interaction term between eCRF and sex (eCRF*sex) using the likelihood ratio test (LRT).

Several sensitivity analyses were conducted. First, excluding the first three years of follow-up to address reverse causality. Second, analyzing eCRF tertiles separately to assess the robustness of our results from the ACLS classification. Third, conducting multiple imputations using chained equations (MICE) under the assumption that data were missing at random. Here, we imputed 20 datasets for nine covariates with missing values, including sitting time, smoking, alcohol consumption, education, occupational class, hypertension, diabetes mellitus, BMI, and PA. Fourth, we repeated the analyses using time-varying covariates among participants who took part in both HUNT2 and HUNT3. In this approach, follow-up time was split at the participation date of HUNT3 using the stsplit function in Stata. Information on covariates in Model 1, such as sitting time, smoking, alcohol consumption, family history of cancer, hypertension, and diabetes mellitus was updated from HUNT3, and the other covariates such as education and occupational class were held constant. All analyses were performed using Stata version 18.0 (StataCorp, College Station, TX, USA).

## Supplementary Information

Below is the link to the electronic supplementary material.

Supplementary Material 1

## Data Availability

The datasets used and analyzed during the current study are available from the HUNT Data Access Committee (email: hunt@medisin.ntnu.no) upon reasonable request. The HUNT data access information describes the policy regarding data availability (https://www.ntnu.edu/hunt/data).

## Abbreviations

ACLS: Aerobics Center Longitudinal Study
BMI: Body mass index
CI: Confidence interval
CRF: Cardiorespiratory fitness
eCRF: Estimated cardiorespiratory fitness
HR: Hazard ratio
HUNT: Trøndelag Health Study
LRT: Likelihood ratio test
PA: Physical activity
PH: Proportional hazards
RHR: Resting heart rate
WC: Waist circumference
