# Supplementary material for: Nonexercise estimated cardiorespiratory fitness in relation to incidence of urinary tract, bladder and kidney cancer in the HUNT study
Source: Sci Rep. 2025 Nov 24;15:45231. doi: 10.1038/s41598-025-29410-7 (PMC12749760; doi:10.1038/s41598-025-29410-7)
Supplement: Supplementary file 1 — Supplementary Material 1 [file 41598_2025_29410_MOESM1_ESM.docx]

**Nonexercise Estimated Cardiorespiratory Fitness in Relation to Incidence of Urinary Tract, Bladder and Kidney Cancer in the HUNT Study**

Youssef Khalil MSc ^1 2^*, Yi-Qian Sun PhD ^3 4 5^, Xiao-Mei Mai PhD ^6^

^1^ Department of Neuromedicine and Movement Science, NTNU, Norwegian University of Science and Technology, Trondheim, Norway

^2^ HUNT Center for Molecular and Clinical Epidemiology, Department of Public Health and Nursing, Norwegian University of Science and Technology, Trondheim, Norway

^3^ Department of Clinical and Molecular Medicine, Faculty of Medicine and Health Science, Norwegian University of Science and Technology, Trondheim, Norway

^4^ Department of Pathology, Clinic of Laboratory Medicine, St. Olavs Hospital, Trondheim University Hospital, Trondheim, Norway

^5^ TkMidt-Center for Oral Health Services and Research, Mid-Norway, Trondheim, Norway

^6^ Department of Public Health and Nursing, NTNU, Norwegian University of Science and Technology, Trondheim, Norway

***Corresponding Author:**

Youssef Khalil

HUNT Center for Molecular and Clinical Epidemiology

Department of Public Health and Nursing

Norwegian University of Science and Technology

Trondheim, Norway

Email: [youssef.s.a.m.khalil@ntnu.no](mailto:youssef.s.a.m.khalil@ntnu.no)

**List of Abbreviations**

ACSM (American College of Sports Medicine), CI (Confidence Interval), DM (Diabetes mellitus), eCRF (Estimated Cardiorespiratory Fitness), HR (Hazard Ratio), HUNT (Trøndelag Health Study), PA (Physical Activity), RHR (Resting Heart Rate), WC (Waist Circumference).

**Supplementary Methods**

*Covariates*

We identified our covariates a priori based on existing literature, including age, sex, sitting time, smoking status, alcohol consumption, educational level, occupational class, hypertension, and diabetes mellitus (DM). They were categorized based on prior HUNT studies ^1,2^. Sitting time was grouped as 0–4, 5–7, and ≥8 hours/day ^3^. Smoking status was classified into never smoked, former smoker (<10, 10–20, >20 pack-years), and current smoker (<10, 10–20, >20 pack-years). Alcohol consumption was categorized according to the frequency of intake per month into abstainer, 1–4, or ≥5 times/month. Educational level was grouped into <10, 10–12, or ≥13 years, and the occupational class was divided into seven categories using the Erikson Goldthorpe Portocarero (EGP) system. This classification reflects the socio-economic status of participants, ranking them from the highest class (class I) to the lowest class (class VII) ^4^. Family history of cancer (yes/no) was based on information on whether any of the relatives (parents, siblings, or children) have or have had cancer (yes/no). Hypertension (yes/no) was defined as using antihypertensive medication or systolic/diastolic blood pressure ≥140/90 mmHg. DM status (yes/no) was determined based on answers to the following question: “Have you had, or do you have DM?” ^5^.

***Supplementary Tables***

**Supplementary Table 1. The baseline characteristics of 46,968 participants in the HUNT Study, stratified by eCRF categories in men and women.**

|  | **eCRF categories in men (*n*=23,375)** | | | **eCRF categories in women (*n*=23,593)** | | |
| --- | --- | --- | --- | --- | --- | --- |
|  | **20% low** | **40% medium** | **40% high** | **20% low** | **40% medium** | **40% high** |
| **N** | 4676 | 9351 | 9348 | 4721 | 9437 | 9435 |
| **Body mass index** |  |  |  |  |  |  |
| Normal/Underweight | 320 (6.8%) | 2509 (26.8%) | 5563 (59.5%) | 477 (10.1%) | 3908 (41.4%) | 6885 (73.0%) |
| Overweight | 2226 (47.6%) | 5805 (62.1%) | 3677 (39.3%) | 1728 (36.6%) | 4306 (45.6%) | 2391 (25.3%) |
| Obesity | 2109 (45.1%) | 1028 (11.0%) | 102 (1.1%) | 2454 (52.0%) | 1193 (12.6%) | 152 (1.6%) |
| Unknown | 21 (0.4%) | 9 (0.1%) | 6 (0.1%) | 62 (1.3%) | 30 (0.3%) | 7 (0.1%) |
| **Physical activity levels** |  |  |  |  |  |  |
| Inactive | 2250 (48.1%) | 2647 (28.3%) | 870 (9.3%) | 2618 (55.5%) | 3710 (39.3%) | 1240 (13.1%) |
| Low | 1407 (30.1%) | 2512 (26.9%) | 1392 (14.9%) | 1239 (26.2%) | 2373 (25.1%) | 1719 (18.2%) |
| Moderate | 634 (13.6%) | 2475 (26.5%) | 3805 (40.7%) | 564 (11.9%) | 2084 (22.1%) | 3983 (42.2%) |
| High | 211 (4.5%) | 1067 (11.4%) | 2485 (26.6%) | 99 (2.1%) | 483 (5.1%) | 1180 (12.5%) |
| Unknown | 174 (3.7%) | 650 (7.0%) | 796 (8.5%) | 201 (4.3%) | 787 (8.3%) | 1313 (13.9%) |
| **Sitting time (hours/day)** |  |  |  |  |  |  |
| ≤4 h | 840 (18.0%) | 1979 (21.2%) | 2230 (23.9%) | 1170 (24.8%) | 2506 (26.6%) | 2504 (26.5%) |
| 5–7 h | 1059 (22.6%) | 2240 (24.0%) | 2347 (25.1%) | 1216 (25.8%) | 2440 (25.9%) | 2532 (26.8%) |
| ≥8 h | 1637 (35.0%) | 3131 (33.5%) | 3023 (32.3%) | 1292 (27.4%) | 2591 (27.5%) | 2636 (27.9%) |
| Unknown | 1140 (24.4%) | 2001 (21.4%) | 1748 (18.7%) | 1043 (22.1%) | 1900 (20.1%) | 1763 (18.7%) |
| **Smoking (pack-years)** |  |  |  |  |  |  |
| Never smoked | 1607 (34.4%) | 3536 (37.8%) | 4242 (45.4%) | 2098 (44.4%) | 4316 (45.7%) | 4667 (49.5%) |
| Former smoker <10 | 579 (12.4%) | 1317 (14.1%) | 1310 (14.0%) | 649 (13.7%) | 1387 (14.7%) | 1375 (14.6%) |
| Former smoker 10–20 | 417 (8.9%) | 765 (8.2%) | 554 (5.9%) | 171 (3.6%) | 322 (3.4%) | 271 (2.9%) |
| Former smoker >20 | 361 (7.7%) | 546 (5.8%) | 346 (3.7%) | 61 (1.3%) | 92 (1.0%) | 79 (0.8%) |
| Current smoker <10 | 401 (8.6%) | 821 (8.8%) | 766 (8.2%) | 696 (14.7%) | 1358 (14.4%) | 1312 (13.9%) |
| Current smoker 10–20 | 425 (9.1%) | 811 (8.7%) | 735 (7.9%) | 536 (11.4%) | 1015 (10.8%) | 845 (9.0%) |
| Current smoker >20 | 474 (10.1%) | 823 (8.8%) | 735 (7.9%) | 247 (5.2%) | 461 (4.9%) | 375 (4.0%) |
| Unknown | 412 (8.8%) | 732 (7.8%) | 660 (7.1%) | 263 (5.6%) | 486 (5.1%) | 511 (5.4%) |
| **Alcohol consumption (times/month)** |  |  |  |  |  |  |
| Never | 1203 (25.7%) | 2090 (22.4%) | 2029 (21.7%) | 2313 (49.0%) | 3705 (39.3%) | 3201 (33.9%) |
| 1–4 | 2472 (52.9%) | 4964 (53.1%) | 5078 (54.3%) | 1842 (39.0%) | 4384 (46.5%) | 4615 (48.9%) |
| ≥5 | 738 (15.8%) | 1758 (18.8%) | 1755 (18.8%) | 225 (4.8%) | 686 (7.3%) | 998 (10.6%) |
| Unknown | 263 (5.6%) | 539 (5.8%) | 486 (5.2%) | 341 (7.2%) | 662 (7.0%) | 621 (6.6%) |
| **Education (years)** |  |  |  |  |  |  |
| <10 | 1551 (33.2%) | 2663 (28.5%) | 2134 (22.8%) | 1937 (41.0%) | 3221 (34.1%) | 2583 (27.4%) |
| 10–12 | 1866 (39.9%) | 3795 (40.6%) | 3712 (39.7%) | 1459 (30.9%) | 2869 (30.4%) | 2681 (28.4%) |
| ≥13 | 1117 (23.9%) | 2710 (29.0%) | 3310 (35.4%) | 1207 (25.6%) | 3140 (33.3%) | 3994 (42.3%) |
| Unknown | 142 (3.0%) | 183 (2.0%) | 192 (2.1%) | 118 (2.5%) | 207 (2.2%) | 177 (1.9%) |
| **Occupational** **class** |  |  |  |  |  |  |
| EGP class I | 456 (9.8%) | 998 (10.7%) | 1089 (11.6%) | 122 (2.6%) | 278 (2.9%) | 386 (4.1%) |
| EGP class II | 316 (6.8%) | 816 (8.7%) | 935 (10.0%) | 620 (13.1%) | 1515 (16.1%) | 1745 (18.5%) |
| EGP class III | 252 (5.4%) | 494 (5.3%) | 572 (6.1%) | 938 (19.9%) | 2206 (23.4%) | 2141 (22.7%) |
| EGP class IV | 811 (17.3%) | 1525 (16.3%) | 1441 (15.4%) | 391 (8.3%) | 663 (7.0%) | 592 (6.3%) |
| EGP class V+VI | 609 (13.0%) | 1396 (14.9%) | 1435 (15.4%) | 119 (2.5%) | 256 (2.7%) | 258 (2.7%) |
| EGP class VII | 657 (14.1%) | 1167 (12.5%) | 1039 (11.1%) | 675 (14.3%) | 1136 (12.0%) | 976 (10.3%) |
| Unknown | 1575 (33.7%) | 2955 (31.6%) | 2837 (30.3%) | 1856 (39.3%) | 3383 (35.8%) | 3337 (35.4%) |
| **Family history of cancer** |  |  |  |  |  |  |
| No | 3638 (77.8%) | 7151 (76.5%) | 7133 (76.3%) | 3470 (73.5%) | 6995 (74.1%) | 7071 (74.9%) |
| Yes | 1038 (22.2%) | 2200 (23.5%) | 2215 (23.7%) | 1251 (26.5%) | 2442 (25.9%) | 2364 (25.1%) |
| **Hypertension** |  |  |  |  |  |  |
| No | 1780 (38.1%) | 4676 (50.0%) | 5824 (62.3%) | 2386 (50.5%) | 6095 (64.6%) | 7014 (74.3%) |
| Yes | 2886 (61.7%) | 4665 (49.9%) | 3505 (37.5%) | 2331 (49.4%) | 3334 (35.3%) | 2410 (25.5%) |
| Unknown | 10 (0.2%) | 10 (0.1%) | 19 (0.2%) | 4 (0.1%) | 8 (0.1%) | 11 (0.1%) |
| **Diabetes mellitus** |  |  |  |  |  |  |
| No | 4413 (94.4%) | 9050 (96.8%) | 9130 (97.7%) | 4470 (94.7%) | 9226 (97.8%) | 9311 (98.7%) |
| Yes | 246 (5.3%) | 274 (2.9%) | 174 (1.9%) | 233 (4.9%) | 181 (1.9%) | 97 (1.0%) |
| Unknown | 17 (0.4%) | 27 (0.3%) | 44 (0.5%) | 18 (0.4%) | 30 (0.3%) | 27 (0.3%) |
| **Abbreviation**: eCRF (estimated cardiorespiratory fitness); EGP (Erikson Goldthorpe Portocarero). **Data**: as means (standard deviation) for continuous variables and participant counts (percentages) for categorical variables. eCRF is stratified by sex and age-specific categories: 20% in the low category, 40% in the medium category, and 40% in the high category. **Note:** Due to rounding, percentages may not precisely sum to 100%. | | | | | | |

**Supplementary Table 2. The association between eCRF and the incidence of urinary tract cancers** **after excluding the first three years of follow-up in the HUNT study.**

| **eCRF categories** | **Cases** | **Incidence rate (per 1000 person-years)** | **Crude model** | |  | **Model 1** | |  | **Model 2** | |  |
| --- | --- | --- | --- | --- | --- | --- | --- | --- | --- | --- | --- |
|  |  |  | **HR (95% CI)** | | **P-value for trend** | **HR (95% CI)** | | **P-value for trend** | **HR (95% CI)** | | **P-value for trend** |
| **Total**  **(*n*=46,176)** |  |  |  | |  |  | |  |  | |  |
| 20% low  (*n*=9157) | 148 | 0.95 | 1.00 | [Reference] | < 0.001 | 1.00 | [Reference] | < 0.001 | 1.00 | [Reference] | 0.002 |
| 40% medium  (*n*=18,465) | 266 | 0.83 | 0.85 | (0.69–1.04) |  | 0.87 | (0.71–1.07) |  | 0.86 | (0.69–1.08) |  |
| 40% high  (*n*=18,554) | 191 | 0.58 | 0.60 | (0.48–0.74) |  | 0.65 | (0.52–0.81) |  | 0.65 | (0.49–0.86) |  |
| **Men**  **(*n*=22,886)** |  |  |  | |  |  | |  |  | |  |
| 20% low  (*n*=4525) | 112 | 1.49 | 1.00 | [Reference] | < 0.001 | 1.00 | [Reference] | < 0.001 | 1.00 | [Reference] | 0.001 |
| 40% medium  (*n*=9166) | 190 | 1.21 | 0.78 | (0.62–0.99) |  | 0.81 | (0.64–1.03) |  | 0.78 | (0.60–1.02) |  |
| 40% high  (*n*=9195) | 132 | 0.82 | 0.53 | (0.41–0.68) |  | 0.59 | (0.45–0.76) |  | 0.56 | (0.40–0.79) |  |
| **Women**  **(*n*=23,290)** |  |  |  | |  |  | |  |  | |  |
| 20% low  (*n*=4632) | 36 | 0.45 | 1.00 | [Reference] | 0.18 | 1.00 | [Reference] | 0.26 | 1.00 | [Reference] | 0.86 |
| 40% medium  (*n*=9299) | 76 | 0.46 | 1.02 | (0.68–1.51) |  | 1.02 | (0.68–1.52) |  | 1.13 | (0.72–1.77) |  |
| 40% high  (*n*=9359) | 59 | 0.35 | 0.79 | (0.52–1.19) |  | 0.81 | (0.53–1.24) |  | 0.98 | (0.56–1.73) |  |

**Abbreviations:** CI (confidence interval); eCRF (estimated cardiorespiratory fitness); HR (hazard ratio).
eCRF is stratified by sex and 10-year age-specific categories: 20% in the low category, 40% in the medium category, and 40% in the high category.
**Crude model:** Age is used as the time scale.
**Model 1:** Age is used as the time scale and adjusted for sex (only in total), sitting time, smoking, alcohol consumption, education, occupational class, family history of cancer, hypertension, and diabetes.
**Model 2:** Adjusted for body mass index and physical activity in addition to variables in Model 1.

**Supplementary Table 3. The association between eCRF categories and the incidence of bladder cancer in HUNT study after excluding the first three years of follow-up in the HUNT study.**

| **eCRF categories** | **Cases** | **Incidence rate (per 1000 person-years)** | **Crude model** | |  | **Model 1** | |  |
| --- | --- | --- | --- | --- | --- | --- | --- | --- |
|  |  |  | **HR (95% CI)** | | **P-value for trend** | **HR (95% CI)** | | **P-value for trend** |
| **Men**  **(*n*=22,886)** |  |  |  | |  |  | |  |
| 20% low  (*n*=4525) | 64 | 0.85 | 1.00 | [Reference] | < 0.001 | 1.00 | [Reference] | 0.009 |
| 40% medium  (*n*=9166) | 125 | 0.80 | 0.89 | (0.66–1.20) |  | 0.93 | (0.69–1.27) |  |
| 40% high  (*n*=9195) | 85 | 0.53 | 0.58 | (0.42–0.80) |  | 0.66 | (0.47–0.92) |  |
| **Women**  **(*n*=23,290)** |  |  |  | |  |  | |  |
| 20% low (*n*=4632) | 16 | 0.20 | 1.00 | [Reference] | 0.62 | 1.00 | [Reference] | 0.66 |
| 40% medium (*n*=9299) | 24 | 0.15 | 0.72 | (0.38–1.36) |  | 0.68 | (0.36–1.28) |  |
| 40% high (*n*=9359) | 36 | 0.21 | 1.08 | (0.60–1.94) |  | 1.00 | (0.55–1.86) |  |

**Abbreviations:** CI (confidence interval); eCRF (estimated cardiorespiratory fitness); HR (hazard ratio).
eCRF is stratified by sex and 10-year age-specific categories: 20% in the low category, 40% in the medium category, and 40% in the high category.
**Crude model:** Age is used as the time scale.
**Model 1:** Age is used as the time scale and adjusted for sitting time, smoking, alcohol consumption, education, occupational class, family history of cancer, hypertension, and diabetes.
**Supplementary Table 4. The association between eCRF categories and the incidence of kidney cancer in HUNT study after excluding the first three years of follow-up in the HUNT study.**

| **eCRF categories** | **Cases** | **Incidence rate (per 1000 person-years)** | **Crude model** | |  | **Model 1** | |  |
| --- | --- | --- | --- | --- | --- | --- | --- | --- |
|  |  |  | **HR (95% CI)** | | **P-value for trend** | **HR (95% CI)** | | **P-value for trend** |
| **Total**  **(*n*=46,176)** |  |  |  | |  |  | |  |
| 20% low  (*n*=9157) | 68 | 0.44 | 1.00 | [Reference] | < 0.001 | 1.00 | [Reference] | < 0.001 |
| 40% medium  (*n*=18,465) | 106 | 0.33 | 0.75 | (0.55–1.01) |  | 0.78 | (0.58–1.07) |  |
| 40% high  (*n*=18,554) | 66 | 0.20 | 0.46 | (0.33–0.64) |  | 0.51 | (0.36–0.72) |  |
| **Men**  **(*n*=22,886)** |  |  |  | |  |  | |  |
| 20% low  (*n*=4525) | 48 | 0.64 | 1.00 | [Reference] | < 0.001 | 1.00 | [Reference] | 0.001 |
| 40% medium  (*n*=9166) | 58 | 0.37 | 0.57 | (0.39–0.84) |  | 0.58 | (0.40–0.86) |  |
| 40% high  (*n*=9195) | 45 | 0.28 | 0.44 | (0.29–0.65) |  | 0.47 | (0.31–0.72) |  |
| **Women**  **(*n*=23,290)** |  |  |  | |  |  | |  |
| 20% low (*n*=4632) | 20 | 0.25 | 1.00 | [Reference] | 0.012 | 1.00 | [Reference] | 0.035 |
| 40% medium (*n*=9299) | 48 | 0.29 | 1.16 | (0.69–1.95) |  | 1.22 | (0.71–2.07) |  |
| 40% high (*n*=9359) | 21 | 0.12 | 0.50 | (0.27–0.93) |  | 0.55 | (0.29–1.04) |  |

**Abbreviations:** CI (confidence interval); eCRF (estimated cardiorespiratory fitness); HR (hazard ratio).
eCRF is stratified by sex and 10-year age-specific categories: 20% in the low category, 40% in the medium category, and 40% in the high category.
**Crude model:** Age is used as the time scale.
**Model 1:** Age is used as the time scale and adjusted for sex (only in total), sitting time, smoking, alcohol consumption, education, occupational class, family history of cancer, hypertension, and diabetes.

**Supplementary Table 5. The association between eCRF tertiles and the incidence of urinary tract cancers in the HUNT study.**

| **eCRF tertiles** | **Cases** | **Incidence rate (per 1000 person-years)** | **Crude model** | |  | **Model 1** | |  | **Model 2** | |  |
| --- | --- | --- | --- | --- | --- | --- | --- | --- | --- | --- | --- |
|  |  |  | **HR (95% CI)** | | **P-value for trend** | **HR (95% CI)** | | **P-value for trend** | **HR (95% CI)** | | **P-value for trend** |
| **Total**  **(*n*=46,968)** |  |  |  | |  |  | |  |  | |  |
| low  (*n*=15,659) | 260 | 0.84 | 1.00 | [Reference] | < 0.001 | 1.00 | [Reference] | < 0.001 | 1.00 | [Reference] | 0.001 |
| medium  (*n*=15,657) | 228 | 0.72 | 0.85 | (0.71–1.01) |  | 0.87 | (0.73–1.04) |  | 0.85 | (0.70–1.04) |  |
| high  (*n*=15,652) | 164 | 0.51 | 0.60 | (0.50–0.73) |  | 0.65 | (0.53–0.79) |  | 0.64 | (0.50–0.82) |  |
| **Men**  **(*n*=23,375)** |  |  |  | |  |  | |  |  | |  |
| low  (*n*=7793) | 187 | 1.25 | 1.00 | [Reference] | < 0.001 | 1.00 | [Reference] | < 0.001 | 1.00 | [Reference] | 0.004 |
| medium  (*n*=7793) | 165 | 1.07 | 0.84 | (0.68–1.04) |  | 0.88 | (0.71–1.08) |  | 0.87 | (0.69–1.10) |  |
| high  (*n*=7789) | 116 | 0.73 | 0.58 | (0.46–0.73) |  | 0.64 | (0.51–0.81) |  | 0.64 | (0.47–0.86) |  |
| **Women**  **(*n*=23,593)** |  |  |  | |  |  | |  |  | |  |
| low  (*n*=7866) | 73 | 0.46 | 1.00 | [Reference] | 0.02 | 1.00 | [Reference] | 0.02 | 1.00 | [Reference] | 0.09 |
| medium  (*n*=7864) | 63 | 0.39 | 0.84 | (0.60–1.18) |  | 0.84 | (0.60–1.19) |  | 0.84 | (0.58–1.23) |  |
| high  (*n*=7863) | 48 | 0.29 | 0.65 | (0.45–0.93) |  | 0.65 | (0.45–0.95) |  | 0.66 | (0.41–1.06) |  |

**Abbreviations:** CI (confidence interval); eCRF (estimated cardiorespiratory fitness); HR (hazard ratio).
eCRF is stratified by sex and 10-year age-specific tertiles.
**Crude model:** Age is used as the time scale.
**Model 1:** Age is used as the time scale and adjusted for sex (only in total), sitting time, smoking, alcohol consumption, education, occupational class, family history of cancer, hypertension, and diabetes.
**Model 2:** Adjusted for body mass index and physical activity in addition to variables in Model 1.

**Supplementary Table 6. The association between eCRF tertiles and the incidence of bladder cancer in the HUNT study.**

| **eCRF tertiles** | **Cases** | **Incidence rate (per 1000 person-years)** | **Crude model** | |  | **Model 1** | |  | **Model 2** | |  |
| --- | --- | --- | --- | --- | --- | --- | --- | --- | --- | --- | --- |
|  |  |  | **HR (95% CI)** | | **P-value for trend** | **HR (95% CI)** | | **P-value for trend** | **HR (95% CI)** | | **P-value for trend** |
| **Men**  **(*n*=23,375)** |  |  |  | |  |  | |  |  | |  |
| low  (*n*=7793) | 112 | 0.80 | 1.00 | [Reference] | 0.002 | 1.00 | [Reference] | 0.031 | 1.00 | [Reference] | 0.43 |
| medium  (*n*=7793) | 110 | 0.73 | 0.93 | (0.72–1.21) |  | 0.98 | (0.75–1.28) |  | 1.05 | (0.78–1.41) |  |
| high  (*n*=7789) | 77 | 0.49 | 0.63 | (0.47–0.85) |  | 0.71 | (0.53–0.96) |  | 0.85 | (0.59–1.23) |  |
| **Women**  **(*n*=23,593)** |  |  |  | |  |  | |  |  | |  |
| low  (*n*=7866) | 25 | 0.16 | 1.00 | [Reference] | 0.44 | 1.00 | [Reference] | 0.59 | 1.00 | [Reference] | 0.93 |
| medium  (*n*=7864) | 26 | 0.16 | 1.02 | (0.59–1.77) |  | 0.99 | (0.57–1.72) |  | 0.88 | (0.48–1.61) |  |
| high  (*n*=7863) | 31 | 0.19 | 1.23 | (0.73–2.08) |  | 1.15 | (0.67–1.99) |  | 1.02 | (0.51–2.05) |  |

**Abbreviations:** CI (confidence interval); eCRF (estimated cardiorespiratory fitness); HR (hazard ratio).
eCRF is stratified by sex and 10-year age-specific tertiles.
**Crude model:** Age is used as the time scale.
**Model 1:** Age is used as the time scale and adjusted for sitting time, smoking, alcohol consumption, education, occupational class, family history of cancer, hypertension, and diabetes.
**Model 2:** Adjusted for body mass index and physical activity in addition to variables in Model 1.

**Supplementary Table 7. The association between eCRF tertiles and the incidence of kidney cancer in the HUNT study.**

| **eCRF tertiles** | **Cases** | **Incidence rate (per 1000 person-years)** | **Crude model** | |  | **Model 1** | |  | **Model 2** | |  |
| --- | --- | --- | --- | --- | --- | --- | --- | --- | --- | --- | --- |
|  |  |  | **HR (95% CI)** | | **P-value for trend** | **HR (95% CI)** | | **P-value for trend** | **HR (95% CI)** | | **P-value for trend** |
| **Total**  **(*n*=46,968)** |  |  |  | |  |  | |  |  | |  |
| low  (*n*=15,659) | 118 | 0.38 | 1.00 | [Reference] | < 0.001 | 1.00 | [Reference] | < 0.001 | 1.00 | [Reference] | < 0.001 |
| medium  (*n*=15,657) | 85 | 0.27 | 0.70 | (0.53–0.93) |  | 0.73 | (0.55–0.97) |  | 0.69 | (0.50–0.94) |  |
| high  (*n*=15,652) | 52 | 0.16 | 0.43 | (0.31–0.59) |  | 0.46 | (0.33–0.65) |  | 0.39 | (0.26–0.59) |  |
| **Men**  **(*n=*23,375)** |  |  |  | |  |  | |  |  | |  |
| low  (*n*=7793) | 73 | 0.49 | 1.00 | [Reference] | < 0.001 | 1.00 | [Reference] | 0.002 | 1.00 | [Reference] | < 0.001 |
| medium  (*n*=7793) | 50 | 0.32 | 0.67 | (0.47–0.96) |  | 0.69 | (0.48–0.99) |  | 0.58 | (0.39–0.87) |  |
| high  (*n*=7789) | 37 | 0.23 | 0.49 | (0.33–0.72) |  | 0.53 | (0.35–0.80) |  | 0.36 | (0.22–0.61) |  |
| **Women**  **(*n*=23,593)** |  |  |  | |  |  | |  |  | |  |
| low  (*n*=7866) | 45 | 0.28 | 1.00 | [Reference] | < 0.001 | 1.00 | [Reference] | < 0.001 | 1.00 | [Reference] | 0.017 |
| medium  (*n*=7864) | 35 | 0.22 | 0.76 | (0.49–1.18) |  | 0.78 | (0.49–1.22) |  | 0.84 | (0.51–1.40) |  |
| high  (*n*=7863) | 15 | 0.10 | 0.33 | (0.18–0.59) |  | 0.34 | (0.19–0.62) |  | 0.39 | (0.18–0.81) |  |

**Abbreviations:** CI (confidence interval); eCRF (estimated cardiorespiratory fitness); HR (hazard ratio).
eCRF is stratified by sex and 10-year age-specific tertiles.
**Crude model:** Age is used as the time scale.
**Model 1:** Age is used as the time scale and adjusted for sex (only in total), sitting time, smoking, alcohol consumption, education, occupational class, family history of cancer, hypertension, and diabetes.
**Model 2:** Adjusted for body mass index and physical activity in addition to variables in Model 1.

**Supplementary Table 8. The association between eCRF categories and the incidence of urinary tract cancers in HUNT study, after applying multiple imputations to the covariates.**

| **eCRF categories** | **Cases** | **Incidence rate (per 1000 person-years)** | **Crude model** | |  | **Model 1** | |  | **Model 2** | |  |
| --- | --- | --- | --- | --- | --- | --- | --- | --- | --- | --- | --- |
|  |  |  | **HR (95% CI)** | | **P-value for trend** | **HR (95% CI)** | | **P-value for trend** | **HR (95% CI)** | | **P-value for trend** |
| **Total**  **(*n*=46,968)** |  |  |  | |  |  | |  |  | |  |
| 20% low  (*n*=9397) | 161 | 0.88 | 1.00 | [Reference] | < 0.001 | 1.00 | [Reference] | < 0.001 | 1.00 | [Reference] | < 0.001 |
| 40% medium  (*n*=18,788) | 287 | 0.76 | 0.85 | (0.70–1.03) |  | 0.87 | (0.71–1.05) |  | 0.83 | (0.67–1.03) |  |
| 40% high  (*n*=18,783) | 204 | 0.53 | 0.60 | (0.48–0.73) |  | 0.63 | (0.51–0.79) |  | 0.60 | (0.46–0.80) |  |
| **Men**  **(*n*=23,375)** |  |  |  | |  |  | |  |  | |  |
| 20% low  (*n*=4676) | 120 | 1.35 | 1.00 | [Reference] | < 0.001 | 1.00 | [Reference] | < 0.001 | 1.00 | [Reference] | < 0.001 |
| 40% medium  (*n*=9351) | 206 | 1.12 | 0.80 | (0.64–1.00) |  | 0.83 | (0.66–1.04) |  | 0.78 | (0.60–1.00) |  |
| 40% high  (*n*=9348) | 142 | 0.75 | 0.54 | (0.42–0.69) |  | 0.59 | (0.46–0.76) |  | 0.54 | (0.39–0.74) |  |
| **Women**  **(*****n*=23,593)** |  |  |  | |  |  | |  |  | |  |
| 20% low (*n*=4721) | 41 | 0.43 | 1.00 | [Reference] | 0.10 | 1.00 | [Reference] | 0.12 | 1.00 | [Reference] | 0.44 |
| 40% medium (*n*=9437) | 81 | 0.42 | 0.96 | (0.66–1.40) |  | 0.96 | (0.65–1.40) |  | 1.00 | (0.65–1.53) |  |
| 40% high (*n*=9435) | 62 | 0.31 | 0.74 | (0.50–1.10) |  | 0.74 | (0.49–1.12) |  | 0.83 | (0.48–1.41) |  |

**Abbreviations:** CI (confidence interval); eCRF (estimated cardiorespiratory fitness); HR (hazard ratio).
eCRF is stratified by sex and 10-year age-specific categories: 20% in the low category, 40% in the medium category, and 40% in the high category.
**Crude model:** Age is used as the time scale.
**Model 1:** Age is used as the time scale and adjusted for sex (only in total), sitting time, smoking, alcohol consumption, education, occupational class, family history of cancer, hypertension, and diabetes.
**Model 2:** Adjusted for body mass index and physical activity in addition to variables in Model 1.

**Supplementary Table 9. The association between eCRF categories and the incidence of bladder cancer in HUNT study, after applying multiple imputations to the covariates.**

| **eCRF categories** | **Cases** | **Incidence rate (per 1000 person-years)** | **Crude model** | |  | **Model 1** | |  | **Model 2** | |  |
| --- | --- | --- | --- | --- | --- | --- | --- | --- | --- | --- | --- |
|  |  |  | **HR (95% CI)** | | **P-value for trend** | **HR (95% CI)** | | **P-value for trend** | **HR (95% CI)** | | **P-value for trend** |
| **Men**  **(*n*=23,375)** |  |  |  | |  |  | |  |  | |  |
| 20% low  (*n*=4676) | 71 | 0.80 | 1.00 | [Reference] | < 0.001 | 1.00 | [Reference] | 0.006 | 1.00 | [Reference] | 0.056 |
| 40% medium  (*n*=9351) | 135 | 0.73 | 0.88 | (0.66–1.17) |  | 0.92 | (0.69–1.23) |  | 0.90 | (0.65–1.24) |  |
| 40% high  (*n*=9348) | 93 | 0.49 | 0.59 | (0.43–0.80) |  | 0.66 | (0.48–0.91) |  | 0.68 | (0.46–1.03) |  |
| **Women**  **(*n*=23,593)** |  |  |  | |  |  | |  |  | |  |
| 20% low (*n*=4721) | 18 | 0.19 | 1.00 | [Reference] | 0.63 | 1.00 | [Reference] | 0.82 | 1.00 | [Reference] | 0.90 |
| 40% medium (*n*=9437) | 26 | 0.13 | 0.70 | (0.39–1.28) |  | 0.65 | (0.35–1.19) |  | 0.55 | (0.28–1.06) |  |
| 40% high (*n*=9435) | 38 | 0.19 | 1.03 | (0.59–1.81) |  | 0.95 | (0.53–1.70) |  | 0.80 | (0.37–1.73) |  |

**Abbreviations:** CI (confidence interval); eCRF (estimated cardiorespiratory fitness); HR (hazard ratio).
eCRF is stratified by sex and 10-year age-specific categories: 20% in the low category, 40% in the medium category, and 40% in the high category.
**Crude model:** Age is used as the time scale.
**Model 1:** Age is used as the time scale and adjusted for sitting time, smoking, alcohol consumption, education, occupational class, family history of cancer, hypertension, and diabetes.
**Model 2:** Adjusted for body mass index and physical activity in addition to variables in Model 1.

**Supplementary Table 10. The association between eCRF categories and the incidence of kidney cancer in HUNT study, after applying multiple imputations to the covariates.**

| **eCRF categories** | **Cases** | **Incidence rate (per 1000 person-years)** | **Crude model** | |  | **Model 1** | |  | **Model 2** | |  |
| --- | --- | --- | --- | --- | --- | --- | --- | --- | --- | --- | --- |
|  |  |  | **HR (95% CI)** | | **P-value for trend** | **HR (95% CI)** | | **P-value for trend** | **HR (95% CI)** | | **P-value for trend** |
| **Total**  **(*n*=46,968)** |  |  |  | |  |  | |  |  | |  |
| 20% low  (*n*=9397) | 71 | 0.39 | 1.00 | [Reference] | < 0.001 | 1.00 | [Reference] | < 0.001 | 1.00 | [Reference] | < 0.001 |
| 40% medium  (*n*=18,788) | 115 | 0.30 | 0.78 | (0.58–1.05) |  | 0.81 | (0.60–1.09) |  | 0.79 | (0.57–1.12) |  |
| 40% high  (*n*=18,783) | 69 | 0.18 | 0.47 | (0.33–0.65) |  | 0.51 | (0.36–0.71) |  | 0.45 | (0.29–0.71) |  |
| **Men**  **(*n*=23,375)** |  |  |  | |  |  | |  |  | |  |
| 20% low  (*n*=4676) | 49 | 0.55 | 1.00 | [Reference] | < 0.001 | 1.00 | [Reference] | 0.001 | 1.00 | [Reference] | < 0.001 |
| 40% medium  (*n*=9351) | 64 | 0.35 | 0.63 | (0.43–0.91) |  | 0.64 | (0.44–0.93) |  | 0.55 | (0.36–0.84) |  |
| 40% high  (*n*=9348) | 47 | 0.25 | 0.45 | (0.30–0.68) |  | 0.49 | (0.32–0.74) |  | 0.33 | (0.19–0.58) |  |
| **Women**  **(*n*=23,593)** |  |  |  | |  |  | |  |  | |  |
| 20% low (*n*=4721) | 22 | 0.23 | 1.00 | [Reference] | 0.008 | 1.00 | [Reference] | 0.02 | 1.00 | [Reference] | 0.33 |
| 40% medium (*n*=9437) | 51 | 0.26 | 1.13 | (0.68–1.86) |  | 1.18 | (0.71–1.96) |  | 1.40 | (0.79–2.48) |  |
| 40% high (*n*=9435) | 22 | 0.11 | 0.49 | (0.27–0.88) |  | 0.52 | (0.28–0.96) |  | 0.70 | (0.32–1.53) |  |

**Abbreviations:** CI (confidence interval); eCRF (estimated cardiorespiratory fitness); HR (hazard ratio).
eCRF is stratified by sex and 10-year age-specific categories: 20% in the low category, 40% in the medium category, and 40% in the high category.
**Crude model:** Age is used as the time scale.
**Model 1:** Age is used as the time scale and adjusted for sex (only in total), sitting time, smoking, alcohol consumption, education, occupational class, family history of cancer, hypertension, and diabetes.
**Model 2:** Adjusted for body mass index and physical activity in addition to variables in Model 1.

**Supplementary Table 11. The association between eCRF categories and the incidence of urinary tract cancers in HUNT study with time-varying covariates.**

| **eCRF categories** | **Cases** | **Incidence rate (per 1000 person-years)** | **Crude model** | |  | **Model 1** | |  | **Model with time-varying covariates** | |  |
| --- | --- | --- | --- | --- | --- | --- | --- | --- | --- | --- | --- |
|  |  |  | **HR (95% CI)** | | **P-value for trend** | **HR (95% CI)** | | **P-value for trend** | **HR (95% CI)** | | **P-value for trend** |
| **Total**  **(*n*=46,968)** |  |  |  | |  |  | |  |  | |  |
| 20% low  (*n*=9397) | 161 | 0.88 | 1.00 | [Reference] | < 0.001 | 1.00 | [Reference] | < 0.001 | 1.00 | [Reference] | < 0.001 |
| 40% medium  (*n*=18,788) | 287 | 0.76 | 0.85 | (0.70–1.03) |  | 0.87 | (0.71–1.05) |  | 0.86 | (0.71–1.04) |  |
| 40% high  (*n*=18,783) | 204 | 0.53 | 0.60 | (0.48–0.73) |  | 0.64 | (0.51–0.79) |  | 0.65 | (0.52–0.80) |  |
| **Men**  **(*n*=23,375)** |  |  |  | |  |  | |  |  | |  |
| 20% low  (*n*=4676) | 120 | 1.35 | 1.00 | [Reference] | < 0.001 | 1.00 | [Reference] | < 0.001 | 1.00 | [Reference] | < 0.001 |
| 40% medium  (*n*=9351) | 206 | 1.12 | 0.80 | (0.64–1.00) |  | 0.83 | (0.66–1.04) |  | 0.81 | (0.65–1.02) |  |
| 40% high  (*n*=9348) | 142 | 0.75 | 0.54 | (0.42–0.69) |  | 0.59 | (0.46–0.76) |  | 0.59 | (0.46–0.76) |  |
| **Women**  **(*n*=23,593)** |  |  |  | |  |  | |  |  | |  |
| 20% low (*n*=4721) | 41 | 0.43 | 1.00 | [Reference] | 0.11 | 1.00 | [Reference] | 0.12 | 1.00 | [Reference] | 0.36 |
| 40% medium (*n*=9437) | 81 | 0.42 | 0.96 | (0.66–1.40) |  | 0.95 | (0.65–1.39) |  | 1.00 | (0.69–1.45) |  |
| 40% high (*n*=9435) | 62 | 0.31 | 0.74 | (0.50–1.10) |  | 0.74 | (0.49–1.11) |  | 0.84 | (0.56–1.26) |  |

**Abbreviations:** CI (confidence interval); eCRF (estimated cardiorespiratory fitness); HR (hazard ratio).
eCRF is stratified by sex and 10-year age-specific categories: 20% in the low category, 40% in the medium category, and 40% in the high category.
**Crude model:** Age is used as the time scale.
**Model 1:** Age is used as the time scale and adjusted for sex (only in total), sitting time, smoking, alcohol consumption, education, occupational class, family history of cancer, hypertension, and diabetes.
**Model with time-varying covariates:** Adjusted for the same covariates in Model 1, with handling sitting time, smoking, alcohol consumption, family history of cancer, hypertension, and diabetes as time-varying covariates based on repeated measurements during follow-up, while keeping education and occupational class unchanged.

**Supplementary Table 12. The association between eCRF categories and the incidence of bladder cancer in HUNT study with time-varying covariates.**

| **eCRF categories** | **Cases** | **Incidence rate (per 1000 person-years)** | **Crude model** | |  | **Model 1** | |  | **Model with time-varying covariates** | |  |
| --- | --- | --- | --- | --- | --- | --- | --- | --- | --- | --- | --- |
|  |  |  | **HR (95% CI)** | | **P-value for trend** | **HR (95% CI)** | | **P-value for trend** | **HR (95% CI)** | | **P-value for trend** |
| **Men**  **(*n*=23,375)** |  |  |  | |  |  | |  |  | |  |
| 20% low  (*n*=4676) | 71 | 0.80 | 1.00 | [Reference] | < 0.001 | 1.00 | [Reference] | 0.006 | 1.00 | [Reference] | 0.028 |
| 40% medium  (*n*=9351) | 135 | 0.73 | 0.88 | (0.66–1.17) |  | 0.92 | (0.69–1.23) |  | 0.95 | (0.71–1.28) |  |
| 40% high  (*n*=9348) | 93 | 0.49 | 0.59 | (0.43–0.80) |  | 0.66 | (0.48–0.90) |  | 0.72 | (0.52–0.98) |  |
| **Women**  **(*n*=23,593)** |  |  |  | |  |  | |  |  | |  |
| 20% low (*n*=4721) | 18 | 0.19 | 1.00 | [Reference] | 0.63 | 1.00 | [Reference] | 0.87 | 1.00 | [Reference] | 0.49 |
| 40% medium (*n*=9437) | 26 | 0.13 | 0.70 | (0.39–1.28) |  | 0.64 | (0.35–1.18) |  | 0.77 | (0.42–1.40) |  |
| 40% high (*n*=9435) | 38 | 0.19 | 1.03 | (0.59–1.81) |  | 0.93 | (0.52–1.66) |  | 1.12 | (0.63–2.01) |  |

**Abbreviations:** CI (confidence interval); eCRF (estimated cardiorespiratory fitness); HR (hazard ratio).
eCRF is stratified by sex and 10-year age-specific categories: 20% in the low category, 40% in the medium category, and 40% in the high category.
**Crude model:** Age is used as the time scale.
**Model 1:** Age is used as the time scale and adjusted for sitting time, smoking, alcohol consumption, education, occupational class, family history of cancer, hypertension, and diabetes.
**Model with time-varying covariates:** Adjusted for the same covariates in Model 1, with handling sitting time, smoking, alcohol consumption, family history of cancer, hypertension, and diabetes as time-varying covariates based on repeated measurements during follow-up, while keeping education and occupational class unchanged.

**Supplementary Table 13. The association between eCRF categories and the incidence of kidney cancer in HUNT study with time-varying covariates.**

| **eCRF categories** | **Cases** | **Incidence rate (per 1000 person-years)** | **Crude model** | |  | **Model 1** | |  | **Model with time-varying covariates** | |  |
| --- | --- | --- | --- | --- | --- | --- | --- | --- | --- | --- | --- |
|  |  |  | **HR (95% CI)** | | **P-value for trend** | **HR (95% CI)** | | **P-value for trend** | **HR (95% CI)** | | **P-value for trend** |
| **Total**  **(*n*=46,968)** |  |  |  | |  |  | |  |  | |  |
| 20% low  (*n*=9397) | 71 | 0.39 | 1.00 | [Reference] | < 0.001 | 1.00 | [Reference] | < 0.001 | 1.00 | [Reference] | < 0.001 |
| 40% medium  (*n*=18,788) | 115 | 0.30 | 0.78 | (0.58–1.05) |  | 0.81 | (0.60–1.10) |  | 0.79 | (0.59–1.06) |  |
| 40% high  (*n*=18,783) | 69 | 0.18 | 0.47 | (0.33–0.65) |  | 0.51 | (0.36–0.72) |  | 0.51 | (0.36–0.72) |  |
| **Men**  **(*n*=23,375)** |  |  |  | |  |  | |  |  | |  |
| 20% low  (*n*=4676) | 49 | 0.55 | 1.00 | [Reference] | < 0.001 | 1.00 | [Reference] | 0.001 | 1.00 | [Reference] | 0.001 |
| 40% medium  (*n*=9351) | 64 | 0.35 | 0.63 | (0.43–0.91) |  | 0.63 | (0.43–0.92) |  | 0.64 | (0.44–0.93) |  |
| 40% high  (*n*=9348) | 47 | 0.25 | 0.45 | (0.30–0.68) |  | 0.49 | (0.32–0.74) |  | 0.51 | (0.34–0.77) |  |
| **Women**  **(*n*=23,593)** |  |  |  | |  |  | |  |  | |  |
| 20% low (*n*=4721) | 22 | 0.23 | 1.00 | [Reference] | 0.008 | 1.00 | [Reference] | 0.02 | 1.00 | [Reference] | 0.019 |
| 40% medium (*n*=9437) | 51 | 0.26 | 1.13 | (0.68–1.86) |  | 1.17 | (0.71–1.95) |  | 1.09 | (0.67–1.79) |  |
| 40% high (*n*=9435) | 22 | 0.11 | 0.49 | (0.27–0.88) |  | 0.52 | (0.28–0.96) |  | 0.51 | (0.28–0.92) |  |

**Abbreviations:** CI (confidence interval); eCRF (estimated cardiorespiratory fitness); HR (hazard ratio).
eCRF is stratified by sex and 10-year age-specific categories: 20% in the low category, 40% in the medium category, and 40% in the high category.
**Crude model:** Age is used as the time scale.
**Model 1:** Age is used as the time scale and adjusted for sex (only in total), sitting time, smoking, alcohol consumption, education, occupational class, family history of cancer, hypertension, and diabetes.
**Model with time-varying covariates:** Adjusted for the same covariates in Model 1, with handling sitting time, smoking, alcohol consumption, family history of cancer, hypertension, and diabetes as time-varying covariates based on repeated measurements during follow-up, while keeping education and occupational class unchanged.


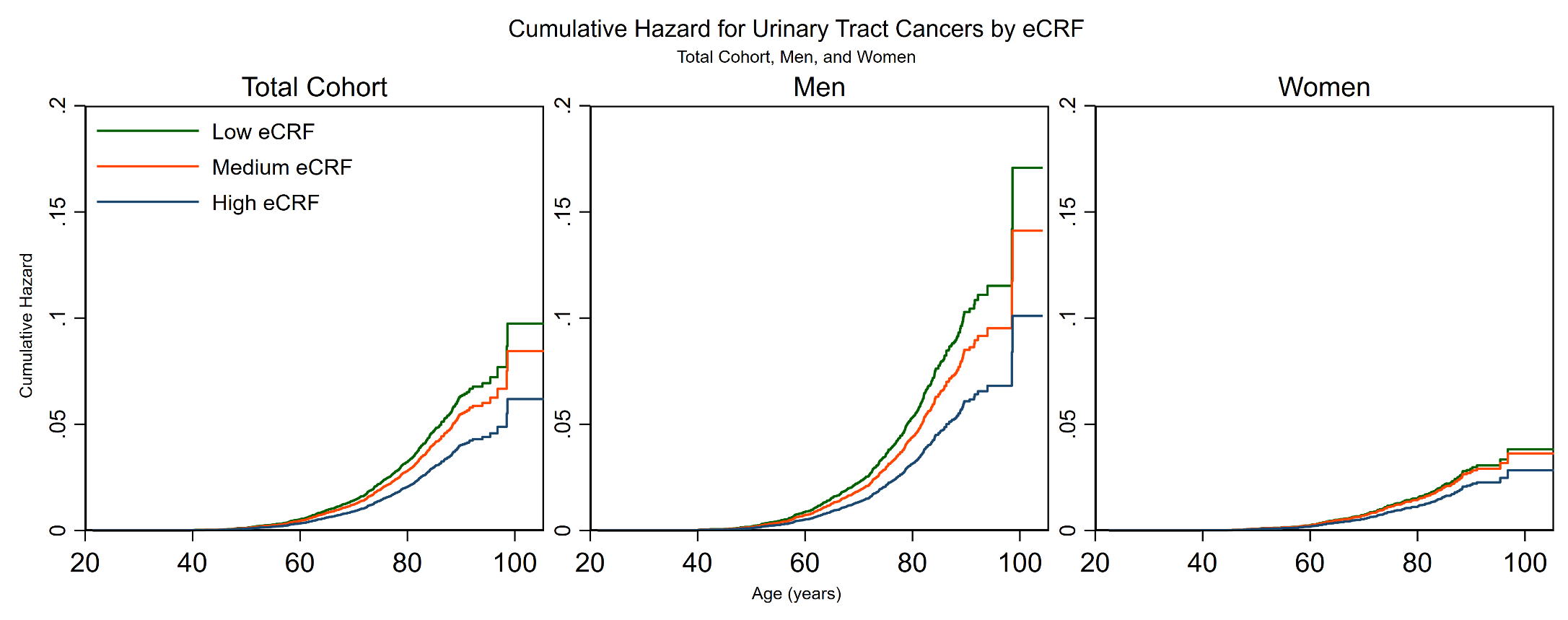


**Supplementary Figure 1.** Cumulative cause-specific hazard curves for urinary tract cancers by estimated cardiorespiratory fitness (eCRF).

Cumulative cause-specific hazard curves for urinary tract cancers are shown for the total cohort (left), men (center), and women (right), stratified by levels of estimated cardiorespiratory fitness (eCRF): low (green), medium (red), and high (blue). Curves were derived from cause-specific Cox proportional hazards models, with age as the time scale. The plots show a lower cumulative hazard in the high eCRF group, especially in the total cohort and among men.

**References**

1 Wang, J., Mai, X.-M. & Sun, Y.-Q. Estimated cardiorespiratory fitness in relation to overall, breast and prostate cancer incidence: the Norwegian HUNT study. *Annals of Epidemiology* **77**, 103–109 (2023). <https://doi.org/https://doi.org/10.1016/j.annepidem.2022.11.008>

2 Garnvik, L. E. *et al.* Estimated Cardiorespiratory Fitness and Risk of Atrial Fibrillation: The Nord-Trøndelag Health Study. *Med Sci Sports Exerc* **51**, 2491–2497 (2019). <https://doi.org/10.1249/mss.0000000000002074>

3 Jiang, L. *et al.* Prolonged Sitting, Its Combination With Physical Inactivity and Incidence of Lung Cancer: Prospective Data From the HUNT Study. *Frontiers in Oncology* **9** (2019). <https://doi.org/10.3389/fonc.2019.00101>

4 Krokstad, S., Ringdal, K. & Westin, S. Classifying people by social class in population based health surveys: Two methods compared. *Norsk Epidemiologi* **12**, 19–25 (2009). <https://doi.org/10.5324/nje.v12i1.501>

5 Midthjell, K., Holmen, J., Bjørndal, A. & Lund-Larsen, G. Is questionnaire information valid in the study of a chronic disease such as diabetes? The Nord-Trøndelag diabetes study. *Journal of Epidemiology and Community Health* **46**, 537–542 (1992). <https://doi.org/10.1136/jech.46.5.537>
